# The INSPIRED study: a randomised controlled trial of the Whole Person Model of disease self-management for people with type 2 diabetes

**DOI:** 10.1186/1471-2458-14-134

**Published:** 2014-02-08

**Authors:** David M Clarke, Donita E Baird, Dinali N Perera, Virginia L Hagger, Helena J Teede

**Affiliations:** 1 Department of Psychological Medicine, Monash University and Monash Health, Level 3, P Block, Monash Medical Centre, 246 Clayton Rd, Clayton, 3168 Melbourne, VIC, Australia; 2 Diabetes Australia Victoria, Melbourne, Australia; 3 Monash Centre for Health Research and Implementation, School of Public Health and Preventative Medicine, Monash University and Diabetes Unit, Monash Health, Melbourne, Australia

**Keywords:** Type 2 diabetes mellitus, Diabetes, Self-management, Depression, Support, Resources

## Abstract

**Background:**

The prevalence of type 2 diabetes has increased dramatically in the last decade, and is continuing to rise. It is a chronic condition, often related to obesity, diet and sedentary lifestyles, and can lead to significant health complications, disability and early death. Diabetes is commonly associated with depression, which can impact significantly on a person’s ability to manage their illness and, consequently, on disease outcomes. Disease self-management is fundamental in diabetes and requires support from multiple health professionals and the active participation of the person, including in maintaining a healthy lifestyle. The Whole Person Model was developed in order to integrate emotional and behavioural aspects into a self-management program for people with type 2 diabetes. Here we describe a study designed to test the efficacy of the Whole Person Model of disease self-management in type 2 diabetes.

**Methods/Design:**

In a parallel-group randomised trial, 180 people with type 2 diabetes of between 2–10 years duration will be recruited via invitation through the Australian National Diabetes Services Scheme. Participants will undergo baseline assessment, followed by randomisation to either intervention or control condition. Control participants will receive fact sheets containing key information about diabetes self-management. The intervention group will receive the INSPIRED (Individual Support & Resources for Diabetes) Manual and be assigned a Health Coach. The INSPIRED Manual consists of six modules that provide key information about diabetes and disease management using the Whole Person Model. Engagement is facilitated by interactive tasks and contact with a Health Coach over seven weeks – an introductory face-to-face session, and six subsequent contacts by phone following each module. Follow-up assessments occur at 13 weeks (post-intervention) and 26 weeks. Primary outcomes include blood glucose management (HbA_1c_), weight and mood. Secondary outcomes include level of exercise, confidence to manage diabetes, and psychosocial well-being.

**Discussion:**

The Whole Person Model is designed to enable health professionals to address mood disturbance without pathologizing any disorders and, in the context of the chronic illness, empowering behavior change and self-management. If proven effective, this model will strengthen capacity of the healthcare workforce to foster and support effective diabetes self-management.

**Trial registration:**

Australia and New Zealand Clinical Trials Register, ACTRN12613000391774

## Background

### Diabetes is a chronic disease

Diabetes mellitus is a metabolic disease with serious implications for both the individual and society. There are currently about 220 million people with diabetes worldwide [1], and the number is projected to exceed 366 million by the year 2030 [2]. Type 2 diabetes accounts for 90–95 percent of cases of diabetes. Although, the specific aetiologies of type 2 diabetes are not known, insulin resistance – that is, where insulin is ineffective in the peripheral tissues – seems to be the major factor, in contrast to absolute insulin deficiency seen in type 1 diabetes [3]. Apart from genetic disposition, the main factors associated with risk of type 2 diabetes appear to be diet, inactivity and body weight – essentially lifestyle factors. Obesity, itself, is associated with insulin resistance. And while the prevalence of obesity is increasing in our communities, there are indications also that type 2 diabetes is increasingly developing earlier in life [4,5]. Type 2 diabetes develops insidiously and often goes undiagnosed for many years because of lack of symptoms in the early stages. It is estimated that up to 6.3 million Americans have undiagnosed diabetes [6].

Diabetes, if undetected or poorly controlled, can result in serious long-term health problems. These include diabetic neuropathy, retinopathy, peripheral vascular disease, renal failure, heart disease and stroke [1]. Worldwide, 2.9 million deaths per year are directly attributable to diabetes, equivalent to 5.2% of world all-cause mortality [7]. Excess mortality attributable to diabetes was over 8% in the U.S.A, Canada and Middle East [7]. Type 2 diabetes is projected to be the leading specific cause of disease burden in Australia for males and second for females by the year 2023 [8].

### Depression and diabetes

Depression is common in people with type 2 diabetes with reported prevalence rates between 9% and 27% [9-11]. This level is about twice as high as that found in the non-diabetic population [12]. The evidence suggests that depression is an independent risk factor for diabetes, with depressed adults having a 37% increased risk of developing type 2 diabetes, compared with those who are not depressed [13]. Clearly there are behavioral factors to partly explain this - for example, depressed people are more likely to be overweight [14], and to exercise less [15]. In addition there are biological factors at play. Potential pathophysiological mechanisms include effects of stress and depression acting via cortisol to stimulate glucose production, increase lipolysis and circulating free fatty acids, decrease insulin secretion, and decrease sensitivity to insulin [16]. Furthermore, depression is associated with increased activity of the pro-inflammatory pathway, and inflammation, also, is associated with insulin resistance [17].

These same mechanisms operate in established diabetes. And in addition to the above, depression interferes with disease management. Depression, and its accompanying feelings of negativity, pessimism, and hopelessness, impairs attention to treatment, diet, exercise etc. [18]. It is very easy for helplessness to set in. Even low levels of depressive symptoms have been shown to be associated with non-adherence to diabetes self-care [19]. Depressive symptoms markedly impair quality of life in both generic and diabetes-specific domains [20] and is associated with higher blood glucose levels [21,22], greater insulin resistance [23], and impaired metabolic control [24]. This, in turn results in more diabetes-related medical complications and higher mortality [25]. Finally, we can note that the presence of depression is associated with an increased cost of diabetes care by 50% [26].

### Chronic disease self-management and the Whole Person Model

The treatment of type 2 diabetes includes weight management, physical activity and dietary modification, together with glucose-lowering medication. To minimize the risk of complications, and to maximize health, regular medical follow-up is required to monitor disease status. This involves regular physical examination (including of feet and eyes), and monitoring of HbA_1c_ and other metabolic risk factors [3]. People with diabetes require a range of services, including primary care physicians, endocrinologists, diabetes educators, dieticians, optometrists and podiatrists; and depending on need, this might include psychiatrists, psychologists, physiotherapists, ophthalmologists, nephrologists, cardiologists and social workers. This can become a full-time occupation for those affected by complicated diabetes and, increasingly, it is recognized that a person with diabetes not only has to manage their illness and comorbidities, emotional distress and lifestyle factors, but also to navigate a complicated health care system, which is not always patient-focused.

Chronic diabetes self-management interventions, including education programs and individual coaching support have been shown to have moderate effectiveness, and vary in their level of intensity [27,28]. Specifically those interventions which incorporate behavioural strategies, and/or ongoing coaching support with a health professional have demonstrated sustained improved outcomes [27,28].

The Diabetes Attitudes Wishes and Needs (DAWN) study [29], a large and ongoing international prospective study of people with diabetes, found the following actions to be important in improving health and quality-of-life for people with diabetes:

•Enhance communications between people with diabetes and healthcare providers

•Promote team-based diabetes care

•Promote active self-management

•Overcome emotional barriers to effective therapy

•Enable better psychological care for people with diabetes.

The Whole Person Model was developed by Baird and Clarke [30,31], within a framework of disease self-management, as a mechanism to integrate all these actions. It essentially uses a cognitive behavioural frame, although, in traditional cognitive behavioural therapy (CBT), less emphasis is usually given to the physical components. In the Whole Person Model, it is understood that how individuals feel (sad, anxious, angry or determined) will affect how they think and behave, including with respect to health related behaviours. These in turn affect health, through inadequate adherence to treatment plans and a healthy lifestyle.

The Whole Person Model approach to self-management aims to provide a health promotion self-management program to enable consumers and health professionals to work together and engage in all aspects of their diabetes management. In addition to providing information about the condition and management strategies, it focuses on enabling health promotion strategies to provide the “*how”* to putting these into place. The program anticipates potential barriers, supports strategies to address these barriers and looks at the emotional impact of the condition. It is designed to provide a useful vehicle for addressing diabetes management from the consumer’s perspective. Although not attempting to specifically diagnose or treat depression or anxiety disorders, it does explore emotional responses in the context of diabetes and its management – 'diabetes-specific distress’ [32]. In this way, the worries, frustrations and helplessness that so often arise with diabetes management are targeted, and support is provided to promote active participation and optimal self-management. In this context, we aim to apply the Whole Person Model in a health promotion, self-management program to improve health related behaviours, health outcomes and mood for those with diabetes, and here, in this manuscript, we describe the study protocol for the INSPIRED (Individual Support & Resources for Diabetes) project.

## Methods/Design

### Study aims and hypotheses

The study aims to evaluate, through a parallel-group randomised controlled trial, the efficacy of a type 2 diabetes self-management program, including a resource consisting of the INSPIRED Manual facilitated by seven weekly sessions with a Health Coach. The supported education intervention promotes wellness by facilitating self-management and is designed to enable those individuals with depression to gain understanding and acknowledgement of their emotional struggles. The Health Coaches are qualified Diabetes Educators, trained in the Whole Person Model. It is hypothesized that, compared to those in the control group, the intervention group will demonstrate improvement in the three primary outcomes of blood glucose management (HbA_1c_), weight and mood. We also hypothesize that improvements will occur in exercise level, ability to manage diabetes and psychosocial well-being.

### Participant recruitment

Participants with type 2 diabetes will be recruited, via invitation, through the National Diabetes Services Scheme which is a large support scheme for people with diabetes. Approximately 1,098,780 Australians with diabetes are registered with the National Diabetes Services Scheme, of which 86% have been diagnosed with type 2 diabetes. Approximately 6% include those who reside in Victoria, meet inclusion criteria and have nominated to receive information about research opportunities related to diabetes. These individuals will be invited to participate in the project. This method of recruitment will be supplemented by advertisement online via the Diabetes Australia, Victoria website. People will indicate their interest by contacting the research team by telephone. Inclusion criteria are: 1) having had diagnosed type 2 diabetes for between 2 and 10 years, 2) aged 18 years or over, 3) a Body Mass Index (BMI) between 20 and 40, 4) not currently on insulin, 5) sufficient speech and reading fluency in English to read and understand the INSPIRED Manual (determined using the Single Item Literacy Screener [33]), and 6) able to give informed consent. People will be excluded if they have significant cognitive impairment or other infirmity which would obviously preclude their participation. If eligible and willing to participate, potential participants will be met by a research assistant, when consent forms will be signed and baseline measures taken.

### Randomisation

The randomisation process will be conducted by an independent statistician. Stata Version 12 [34] statistical package will be used for the random allocation of subjects to either the intervention or the control group. To ensure equal allocation (1:1), block randomisation method will be used. A pre-defined randomised list containing ID numbers and their respective treatment allocation will be provided to the Project Coordinator (DP) who will not be involved in taking measurements, data management or statistical analyses. ID numbers will be assigned to participants in consecutive order to avoid selection bias.

### Intervention

Participants randomised to the control group will receive standard information sheets containing key information about diabetes and diabetes self-management. The information sheets primarily include those which are part of an information series from State/Territory organisation of Diabetes Australia. These will be posted to participants from the study headquarters.

Participants randomised to active treatment will be assigned a trained Health Coach, and will be provided with the INSPIRED Manual during an initial face-to-face meeting with their Health Coach. The INSPIRED Manual provides key information about diabetes and management of diabetes, guidance about diet and exercise, and skills training. Issues of emotions (determined, sad, anxious, angry) are acknowledged and addressed in the context of their disease self-management and their relationships with family and health professionals. This is all designed to facilitate self-management. The specific topics covered have been developed through consumer participation and consultation with key clinical experts and stakeholders (see Table 1 for further intervention details). Using the Whole Person Model, the INSPIRED Manual in combination with the support and guidance of a Health Coach, aims to encourage the reader to consider how their body, their thoughts and feelings, and their health behaviors are all interdependent. This combination aims to help people to be aware of their thinking and learn strategies to improve their mood and make lifestyle changes. An accompanying INSPIRED Journal will also be provided to participants, which assists them to keep track of their medical appointments, health goals and to improve communication with their health professionals.

**Table 1:** Overview of the INSPIRED intervention

**INSPIRED manual content**	**Health coach support**
Diabetes information	Providing weekly support and education about:
	▪ General diabetes
	▪ Diabetes complications
	▪ Hypoglycaemia episodes
	▪ Medication
	▪ Diabetes and driving
	▪ Diabetes and alcohol/smoking
	▪ Importance of self-management
Healthy eating	Providing weekly support and education about:
	▪ Barriers to healthy eating
	▪ Tips for healthy eating and snack choices
	▪ Managing food cravings
	▪ Home-grown experiments
	▪ Emotions and eating
	▪ Eating out
	▪ Reading food labels
Becoming more physically active	Providing weekly support and education about:
	▪ Attitudes about different types of physical activity
	▪ Benefits of physical activity
	▪ Keeping track of your steps using your pedometer
	▪ How to be more physically active
	▪ Physical activity and blood glucose levels
Weight management	Providing weekly support and education about:
	▪ Your Body Mass Index
	▪ Losing weight the healthy way
	▪ Weight loss and maintenance tips
	▪ Healthy eating tips for weight loss
Monitoring your diabetes	Providing weekly support and education about:
	▪ Keeping track of your diabetes using your Journal
	▪ Paying attention to your blood glucose levels
Maintaining contact with your doctor and other health professionals	Providing weekly support and education about:
	▪ Choosing a doctor
	▪ The importance of keeping in regular contact with your doctor
	▪ How to get the most from your doctor’s visit
Family and social support	Providing weekly support and education about:
	▪ Carers and helpers
	▪ Staying connected
	▪ Making friends
Dealing with emotions (i.e., worry, anger)	Providing weekly support and education about:
	▪ Negative thoughts
	▪ Managing distress
	▪ Relaxation
	▪ Reducing feelings of tension and worry – problem solving
	▪ Tips to reduce the impact of anger
The Whole Person Model	Providing weekly support and education about:
	▪ The Whole Person Model
	▪ Considering your body, feelings and thoughts when making an Action Plan
	▪ Examining your thinking and talking back unhelpful thoughts
Toolbox for a healthy life	Providing weekly support and education about:
	▪ What to put in your tool box for a healthy life?
	- Things I am going to try
	- Things I am going to think about
	- Other ideas

Participants will receive seven sessions with a Health Coach over a 7 week period as follows:

•One face-to-face session, involving an orientation to the INSPIRED Manual and the Whole Person Model, conducted at the study headquarters.

•Six weekly phone coaching sessions. During these calls the Health Coach will support the participant working through the INSPIRED Manual, answering questions, helping with goal-setting, and discussing the homework exercises.

### Outcomes assessments

Measures will be assessed at baseline, 13 weeks and 26 weeks.

Primary outcome measures will be blood glucose management (HbA_1c_), weight and mood. Secondary outcome measures include level of exercise, confidence to manage diabetes (self-report) and psychosocial well-being (self-report) using validated tools. The research assistant collecting the data will be uninformed of group allocation.

### Measures

Details about participants, including age, sex, height, education level, employment status and current medications will be obtained at baseline. Weight, waist girth and HbA_1c_, will be measured at each time point (baseline and 13 and 26 weeks). Participants will undergo a blood test at each time point, at Monash Medical Centre, Melbourne, Australia. Normal HbA_1c_ will be assayed on site at Southern Cross Pathology, Monash Medical Centre, Melbourne, Victoria, Australia. Assay instrument is Adams A1C HA-8160 automatic Glycohaemoglobin analyzer. The method is based upon the principles of high performance liquid chromatography (HPLC).

The following validated self-report measures will also be measured at each time point:

• ***Mood:*** The Depression, Anxiety and Stress Scale (DASS) [35] is a 21-item scale that assesses three negative emotional states: Depression, Anxiety and Stress and is shown to have excellent psychometric properties in adults [36].

• ***Experience of demoralisation:*** The Demoralisation Scale [37] is a 24-item self-report scale with a Cronbach’s alpha of 0.96, measuring depression commonly seen in the physically ill – particularly aspects of helplessness, hopelessness and loss of meaning [37,38].

• ***Social support:*** The Multidimensional Scale of Perceived Social Support Scale [39] is a 12-item scale that measures perceived social support from family, friends and significant others. This scale has proven to be psychometrically sound in diverse samples with strong test-retest reliability over a 2–3 month interval (*r* = 0.72 to 0.85), and internal consistencies of the total scale and sub-scales which are high (Cronbach’s Alpha = 0.79 to 0.98) [39].

• ***Self-efficacy:*** Diabetes Empowerment Scale – Short Form [40] is an 8-item self-report scale which is a valid and reliable measure of perceived ability to manage the psychological and social demands and challenges associated with diabetes [40]. Higher mean scores indicate greater level of confidence managing one’s diabetes.

• ***Level of exercise:*** Participants will be asked to record the total number of steps taken over any three days within a seven day period. This will be measured using a Be Active pedometer (Multifunction Pedometer, WWA2026, Reed Switch Technology) provided to them at the initial assessment. A three day period was noted by Tudor-Locke et al. [41] as a sufficient number of days to reliably measure physical activity in adults.

• ***Quality of Life:*** The EuroQol (EQ-5 D) [42] is a brief self-report measure of subjective quality of life. This measure has been used in intervention studies with depressed patients [43], and will allow the calculation of quality-adjusted life years (QALY).

***The Working Alliance Inventory – Short Form, Participant/Therapist Version*** (WAI) is a valid measure of treatment alliance [44] and will be completed by participants in the intervention group following phone sessions at 1, 3 and 6 weeks, and by the Health Coach at these times.

### Study integrity and quality assurance

Ethical approval for this study has been obtained from both the Monash Health Human Research Ethics Committee and the Monash University Human Research Ethics Committee. The study design will be guided by the CONSORT statement [45,46]. Each participant will be screened for eligibility and enrolled if eligible and consenting. To ensure consistency and best practice, participant’s height, weight and girth measurements are taken as per recommendations outlined by the World Health Organization [47]. The randomisation process will be conducted by an independent statistician. Assessments at baseline, 13 and 26 weeks will be carried out by a research assistant who will be uninformed of group membership. All Health Coaches who will be delivering the intervention are qualified Diabetes Educators and trained in the Whole Person Model. As well as the initial training, reflective practice support and supervision will be provided to the Health Coaches. The Project Coordinator will monitor the delivery of the intervention via recordings of phone sessions for the first participant of each Health Coach and random subsequent coaching sessions. Constructive feedback will be provided where necessary, and a fidelity checklist completed. Quality of health coaching will also be assured using measures of treatment alliance. No significant risks to participants are anticipated.

### Sample size justification

Using information from comparative studies, 150 participants are required to detect a between group difference of 0.7% (SD ± 1.5) in HbA_1c_, with 80% power and at an alpha level of 0.05 [48]. Similarly, 150 participants are needed to detect a between group difference in weight of 4.4 kg (SD ± 9.6) with 80% power and at an alpha level of 0.05 [49]. Based on a comparative sample using the DASS [50], at least 116 participants are required to detect a hypothesised reduction of 25% in DASS score (20 to 15, SD ± 9.6). On the basis of this analysis, and assuming a dropout rate of 20% based on prior experience, we estimate that a recruited sample of 180 will be required and be sufficient to give the requisite power.

### Statistical analyses

Continuous variables will be assessed for their distribution and outliers prior to analyses. Variables will be transformed if distributional/linearity assumptions are violated. We will compare group characteristics using student t-test for continuous variables, Chi-square (χ^2^) or Fisher’s exact test for categorical variables. We will conduct univariable and multivariable linear regression analyses adjusting for appropriate covariates and test for interactions. For dichotomous outcomes, logistic regression analyses will be implemented. All analyses are to be conducted on an intention-to-treat basis (ITT). However, we may conduct a sensitivity analysis using the imputation method chained equations (ICE) if we find a disproportionate level of missing values that are assessed as missing at random (MAR). Stata Version 12 [34] will be used for all statistical analyses.

## Discussion

This study will examine the efficacy of a novel model of disease self-management. The novelty is the Whole Person Model, which integrates usual self-management principles with attention to the feelings of the person in a way that does not pathologize depression or anxiety, but seeks to understand it in the context of a person struggling, perhaps, with their disease and management of it. In this program we include access to a trained Health Coach as we have concluded from our experience that this offers necessary support to the person whilst they take in new information, and attempt significant and sometimes difficult, behaviour change. We know that both the integrated model and the support of the Health Coach are critical factors in this change. Such knowledge has informed the design of the study that compares the active intervention with a fairly passive approach of delivery of information in the control group. If shown to be effective, this model will be easily implementable in clinical practice. Diabetes Educators are commonly employed around the world, and the Whole Person Model is operationalized in the INSPIRED Manual. Consequently, the model could easily be widely implemented in a way that would have a huge effect on population health.

## Abbreviations

INSPIRED: Individual support & resources for diabetes; DAWN: Diabetes attitudes wishes and needs; CBT: Cognitive behavioral therapy.

## Competing interests

The authors declare that they have no competing interests.

## Authors’ contributions

DC and DB developed the Whole Person Model. DB did much of the drafting of the INSPIRED Manual. DC, DB, HT, VH designed the study, reviewed the manual and were granted national competitive funding. DC will manage the project, while DP will be Project Coordinator. DC will provide supervision of Health Coaches. DP and DC prepared the first draft of this paper. All authors have contributed to the paper, and all authors have read and approved the final manuscript.

## Pre-publication history

The pre-publication history for this paper can be accessed here:

http://www.biomedcentral.com/1471-2458/14/134/prepub
